# Diet in Sickness and in Health

**Published:** 1896-06

**Authors:** 


**Diet in Sickness and in Health.** By Mrs. ERNEST HART. Pp. xii., 219. London: The Scientific Press, Limited. 1895.

The public and the profession alike will acknowledge that no one is better qualified to write on this topic than Mrs. Ernest Hart. Her book will convey much useful information to lay readers, and the many dietaries founded on good physiology of the latest type will be of aid to the medical practitioner. Sir Henry Thompson, in the preface, speaks highly of the book "because its accomplished authoress has the advantage of possessing not only a remarkable acquaintance with the various branches of medical knowledge, after many years devoted to their study, but also in no less degree that which has been conferred by long culinary and housewifery experience." The great variety of dishes suggested for the diabetic and the gouty renders their necessary limitations by no means irksome.

In a work of this character we are sorry to see several advertisements bound in, even although they are included under the euphemistic title of "Appendix." The book is too good to be thus disfigured.

